# Clinical and scientific advances in neurosurgery

**DOI:** 10.1515/iss-2021-2037

**Published:** 2021-04-27

**Authors:** Gabriele Schackert, Tareq A. Juratli

**Affiliations:** Department of Neurosurgery, Carl Gustav Carus University Hospital, Technische Universität Dresden, Dresden, Germany

This special issue is dedicated to clinical and scientific advances in neurosurgery. It provides insights in the broad spectrum of neurosurgery, spanning topics from the peripheral neuromodulation in paretic patients, to special aspects in the management of skull base tumors as well as advances in the treatment of the degenerative spine. Finally, an outlook on experimental immunotherapeutic strategies, explaining the principles of vaccine-based immunotherapy in brain tumors, is presented.

Two original articles deal with recent advances in neurosurgical techniques. The spastic drop foot, caused after stroke or during the course of multiple sclerosis (MS), limits the patients’ quality of life in a significant dimension. Martin et al. present long-term results after implantation of the ActiGait^®^ system—an implantable peroneal nerve stimulation—at 36-month follow-up. The study includes 27 stroke and six multiple sclerosis patients. Selective electrical stimulation was applied to the fascicles of the peroneal nerve through a four-channel cuff electrode proximal to the knee joint, which contracts the ankle dorsiflexor and everter muscles followed by a balanced dorsiflexion of the foot. Patients have been assessed for gait endurance, speed, risk of fall and quality of life at baseline and 36 months following implantation. Most of the patients reported remarkable improvements of their walking abilities and especially of their daily quality of life. These results highlight the long-lasting effect of the ActiGait^®^ system as an effective treatment of the spastic drop foot.

The degenerative spine disease is a widespread disease with an enormous impact on the professional, the economic, and the social life. Therefore, in the second original article we focused on innovations in spine surgery. Salchow-Gille et al. undertook a study, in which they evaluated software-based pre-surgical simulation effects on device selection and device development. Based on video-fluoroscopic motion recordings and pixel-precise processing of the segmental motion patterns, a software-based surrogate functional model was tested. 253 randomly selected patients, requiring single-level cervical or lumbar implant, were included in the study. Based on the described software-surrogate, the authors concluded that individualized devices might decelerate degeneration of adjacent segments by influencing the intersegmental communication of the motion segments and subsequently improve patient’s outcome.

Tumor surgery is one of the main domains in neurosurgery. We selected for this issue the special problems in craniopharyngioma treatment, which combines several medical disciplines e.g. endocrinology, ophthalmology, and neurosurgery. As in all brain tumor surgeries, the preservation of the neurological functions is the primary goal. The special challenge in craniopharyngioma surgery is the additional preservation of endocrine and visual functions. Fujio et al. discuss in their original article two competing surgical strategies: the trans-sphenoidal and the transcranial approach. The authors assessed the clinical courses of 39 patients with newly diagnosed craniopharyngiomas. Of those, 24 patients underwent transcranial microsurgery and 15 trans-sphenoidal surgery as the initial treatment. Fujio et al. found a low rate of surgical complications and a sufficient tumor control rate in response to the treatment strategy. Moreover, the special problems of preserving the hormonal function are highlighted.

Under malignant brain tumors, glioblastomas play a prominent role. For decades, neuro-oncology research has concentrated on innovative strategies for improving the dismal prognosis of patients with glioblastomas. Immunotherapy, as a potential successful treatment, comes increasingly more into focus. The review by Tietze et al. provides a brief introduction to the principles of vaccine-based immunotherapy and gives an overview of current clinical trials in glioblastoma treatments. These include immune checkpoint inhibitors, genetically modified oncolytic viruses, tumor cell lysate or peptide-pulsed dendritic cell vaccines and peptide vaccines. Despite multimodal therapies, the prognosis of glioblastoma patients is still dismal. Many features contribute to this therapeutic challenge including high intratumoral and intertumoral heterogeneity, resistance to therapy, migration and invasion, and immunosuppression. In parallel, the reader is introduced to the molecular principles of T cell-mediated immunosurveillance and the interrelated immunosuppressive factors that influence the glioblastoma microenvironment in order to understand hurdles affecting the efficacy of glioblastoma vaccines.

We are grateful for all the work the authors have included in this special issue.

